# P-299. Clinical and microbiological characteristics of hospital-onset and community-onset invasive urinary tract infection caused by extended-spectrum β-lactamase-producing *Escherichia coli*

**DOI:** 10.1093/ofid/ofae631.502

**Published:** 2025-01-29

**Authors:** Yusuke Tsuda, Yasufumi Matsumara, Kayoko Hayakawa, Shinya Tsuzuki, Koh Shinohara, Soichi Arasawa, Yasuhiro Tsuchido, Satomi Yukawa, Taro Noguchi, Masaki Yamamoto, Miki Nagao

**Affiliations:** Kyoto University Graduate School of Medicine, Kyoto, Kyoto, Japan; Kyoto University Graduate School of Medicine, Kyoto, Kyoto, Japan; National Center for Global Health and Medicine, Shinjuku-ku, Tokyo, Japan; National Center for Global Health and Medicine, Shinjuku-ku, Tokyo, Japan; Kyoto University Graduate School of Medicine, Kyoto, Kyoto, Japan; Kyoto University Graduate School of Medicine, Kyoto, Kyoto, Japan; Kyoto University Graduate School of Medicine, Kyoto, Kyoto, Japan; Kyoto University Graduate School of Medicine, Kyoto, Kyoto, Japan; Kyoto University Graduate School of Medicine, Kyoto, Kyoto, Japan; Kyoto University Graduate School of Medicine, Kyoto, Kyoto, Japan; Kyoto University Graduate School of Medicine, Kyoto, Kyoto, Japan

## Abstract

**Background:**

In the era of community spread of extended-spectrum β-lactamase-producing *Escherichia coli* (ESBLEC), the differences in clinical and microbiological characteristics between hospital-onset (HO) and community-onset (CO) invasive urinary tract infection (iUTI) caused by ESBLEC have not been well documented in East Asia.

**Figure:**
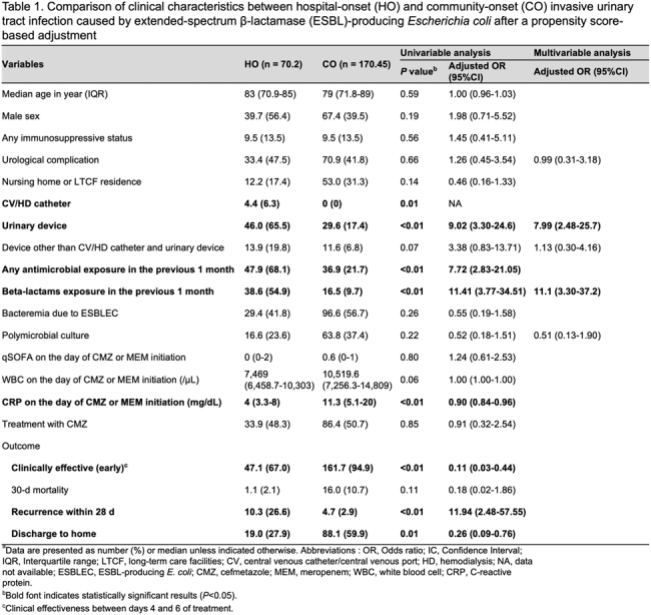

**Methods:**

We conducted a post-hoc analysis of a multicenter, observational study between 2020 and 2021 at 10 facilities across Japan. The inclusion criteria were 1) diagnosis of iUTI, 2) isolation of ESBLEC from urine or blood cultures, and 3) treatment with cefmetazole (CMZ) or meropenem (MEM) as definitive therapy within 96 hours of culture collection and continued for at least 3 days. Patients diagnosed with iUTI within 3 days after admission were assigned to the CO group, while others were assigned to the HO group. Clinical characteristics of the HO and CO groups were compared after reduction of treatment bias between the CMZ and MEM groups using a propensity score-based adjustment. Antimicrobial susceptibility testing and whole-genome sequencing analysis were performed to investigate differences between the HO and CO isolates.

**Figure:**
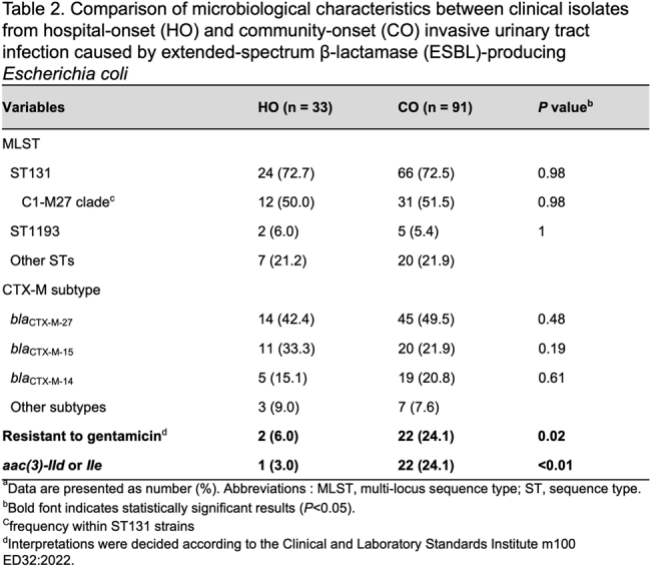

**Results:**

Thirty-two and ninety-five patients were included in the HO and CO groups, respectively.

The clinical and microbiological characteristics were summarized in Tables 1 and 2, respectively.

In multivariable logistic regression analysis, exposure to β-lactam antimicrobials and indwelling urinary catheter were associated with the HO group. Clinical effectiveness in the early treatment period was more frequently observed in the CO group, although the 30-day mortality rates were not significantly different between the HO and CO groups. There was no difference in distributions of sequence types and CTX-M subtypes between the HO and CO isolates. The CO isolates had a higher resistance rate to gentamicin than the HO isolates. Pangenome analysis revealed that *aac(3)-II* (*aac(3)-IId* or *IIe*), which confers resistance to gentamicin, was frequently present in the CO isolates.

**Conclusion:**

Compared to the patients with CO-iUTI by ESBLEC, those with HO-iUTI were characterized by prior exposure to β-lactam antimicrobials and indwelling urinary catheter. The CO isolates were associated with resistance to gentamicin and carriage of *aac(3)-II*.

**Disclosures:**

**All Authors**: No reported disclosures

